# The Anxiety-AFib connection: a systematic review of mental health and arrhythmia interactions

**DOI:** 10.21542/gcsp.2026.28

**Published:** 2026-06-30

**Authors:** Sania Simon, Kavach J. Josh, Vilina H. Gangolli, Irene Sairah Paul, Kedargouda S. Patil, Aneesh Nelivigi, Kevin Thomas Mathew, Amna Shamim, Lakshmi Priya Kopudu Sridharbabu

**Affiliations:** 1 Ivane Javakhishvili Tbilisi State University, Tbilisi, Georgia; 2 Caucasus International University, Tbilisi, Georgia; 3 Tbilisi State Medical University, Tbilisi, Georgia; 4 New Vision University, Tbilisi, Georgia; 5 David Tvildiani Medical University, Tbilisi, Georgia

## Abstract

**Background:** Atrial fibrillation is the most common cardiac arrhythmia, which has effects that goes beyond the heart, often affecting mental health. Over the years, increasing evidence has highlighted a bidirectional connection between anxiety and atrial fibrillation. However, the connection between the two in clinical practice is vague.

**Objective:** This systematic review aims to investigate the relationship between anxiety and atrial fibrillation, including how anxiety might contribute to the onset and relapse of atrial fibrillation, how it may impact the treatment outcomes and its effects on the patient’s overall quality of life.

**Methods:** We conducted an extensive search of PubMed, Scopus, Embase, and Cochrane to systematically identify studies between 2020 and 2025. Adult patients with atrial fibrillation diagnosis who were assessed for anxiety were included using validated scales. A total of 84 articles met the inclusion criteria, with data extracted on anxiety measures, features of atrial fibrillation, clinical impact, and treatment effects.

**Results:** Among the reviewed studies, anxiety was both prevalent in AF patients and significantly associated with poorer clinical outcomes across the majority of included studies, though the strength and nature of associations varied across study designs and populations. Patients with higher levels of anxiety had a higher probability of recurrent atrial fibrillation, increased symptom burden, and lower health-related quality of life. Anxiety also influenced treatment outcomes and adherence, particularly regarding anticoagulation and ablation therapy. Notably, several interventions including catheter ablation and structured patient education were associated with reductions in anxiety and improvements in outcomes. Importantly, one Mendelian randomisation study found no causal relationship between anxiety and AF, while depression and panic disorder showed causal associations, underscoring the need for cautious interpretation of observational findings.

**Conclusion:** Anxiety and atrial fibrillation appear to share a complex, predominantly bidirectional association. While most observational studies support a link between anxiety and worse AF-related outcomes, evidence from Mendelian randomisation does not confirm a causal role for anxiety in AF onset, suggesting that residual confounding may partly account for observational associations. Addressing mental health in AF care is nonetheless clinically important. Incorporating psychological assessment and support into AF management may reduce symptom burden and improve treatment adherence and quality of life. Further research, particularly longitudinal interventional studies using standardised instruments, is required to guide more comprehensive, patient-centred care.

## Introduction

Atrial fibrillation (AF) is the most common sustained cardiac arrhythmia and is associated with substantial morbidity, including stroke, heart failure, and reduced quality of life. The medical community has traditionally classified AF as a condition which stems from structural, electrophysiological, and cardiovascular elements yet new research shows that psychological factors like anxiety play a crucial role in developing AF and in determining its symptoms and disease progression. The occurrence of anxiety disorders reaches high levels in people with cardiovascular diseases, while about 33% of atrial fibrillation patients experience intense anxiety symptoms.

Anxiety may affect the development of AF through several pathways, including activation of the sympathetic nervous system, disruption of hypothalamic-pituitary-adrenal (HPA) axis function, promotion of inflammatory responses, and maladaptive illness beliefs. These mechanisms may collectively worsen cardiac rhythm instability, amplify symptom burden, and impair daily functioning. Conversely, the cardinal symptoms of AF—palpitations, dyspnoea, and dizziness—can themselves provoke or sustain anxiety, suggesting a bidirectional relationship between the two conditions.

Psychocardiology has gained interest, yet the specific connection between anxiety and atrial fibrillation (AF) remains insufficiently defined. Research studies in this area have used different research designs, anxiety measurement instruments, and outcome variables, which prevents researchers from forming unified conclusions. Research studies have not yet conducted a complete assessment of anxiety effects on AF occurrence and recurrence, treatment results, and patient life satisfaction.

## Methods

### Search strategy and selection

An electronic search of several databases such as PubMed, Scopus, Embase, and Cochrane was performed to systematically identify studies between 2020 and April 2025 that met the inclusion criteria. The authors used differing combinations of the following keywords: atrial fibrillation, AFib, anxiety, anxiety disorder, mental health, psychological stress, arrhythmia, recurrence, risk factors, psychocardiology, cardiac arrhythmia and anxiety.

**Figure 1.**
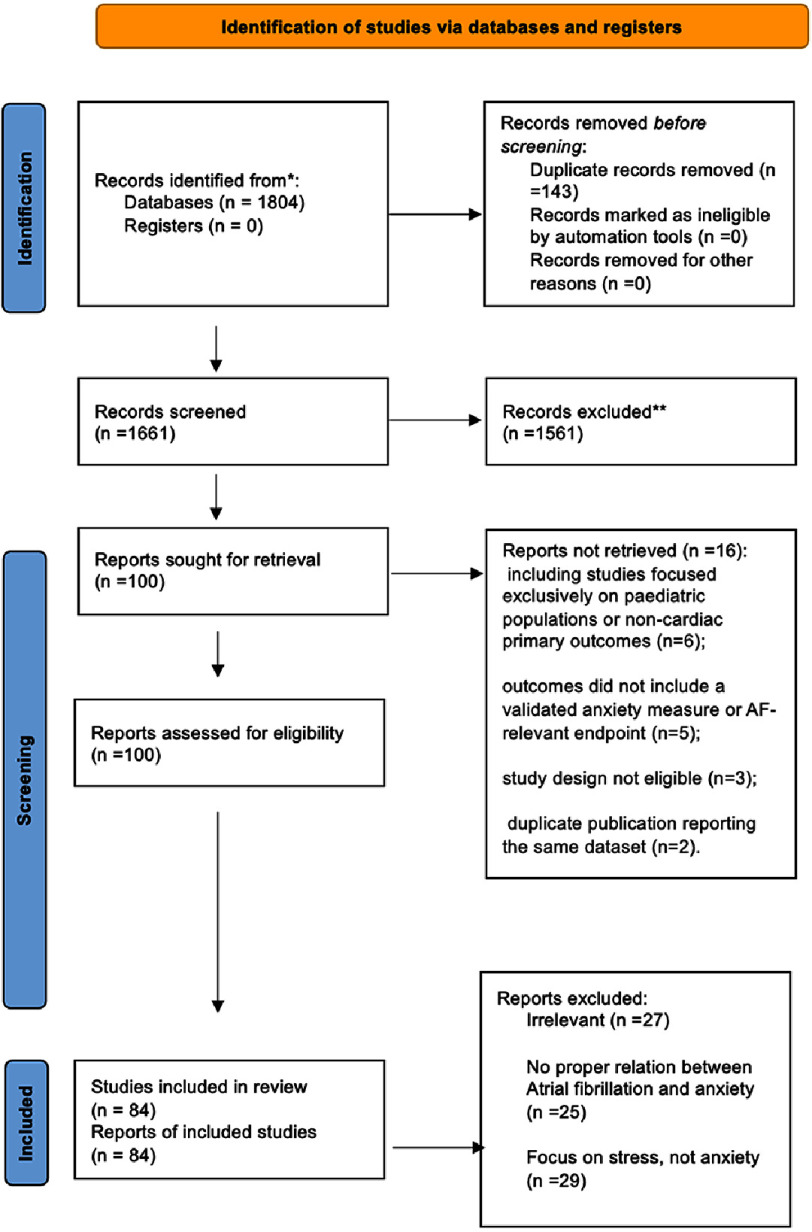
PRISMA 2020 flow diagram for new systematic reviews which included searches of databases and registers only.

Articles were initially collected by a single author and then assessed independently for eligibility by two authors. Title and abstract screening was performed using the systematic review software Rayyan QCRI. Articles meeting the inclusion criteria were further assessed by full-text review by two authors; discrepancies were resolved by a third author. This review was not prospectively registered with PROSPERO. The exact number of articles screened and reasons for exclusion are detailed in the PRISMA 2020 flowchart (Figure 1).

### PICO framework

**Table:** 

P (Population)	Adults (≥18 years) diagnosed with atrial fibrillation (paroxysmal, persistent, or permanent)
I (Intervention / Exposure)	Presence of anxiety disorders or clinically significant anxiety (diagnosed via scales like GAD-7, HADS, etc.)
C (Comparison)	AFib patients without anxiety / General population without AFib / Those without anxiety
O (Outcomes)	Incidence or recurrence of AFib, hospitalizations, symptom burden, treatment outcomes, quality of life

### Eligibility criteria

Studies that were published in English in peer-reviewed journals between 2020 and April 2025 involving adult populations (≥18 years), those assessing anxiety using validated scales (e.g., GAD-7, HADS, Beck Anxiety Inventory), incidence, prevalence, recurrence, or outcomes of atrial fibrillation, with the study designs of cohort, case-control, cross-sectional, or RCTs, were included. All non-English articles, studies focused on paediatric or adolescent populations, non-human studies, case reports, editorials, commentaries, and letters, or those that did not report data on both anxiety and atrial fibrillation (e.g., focused solely on depression, PTSD, or other psychiatric conditions without anxiety) were excluded from the review. A small number of borderline studies were included on the basis that, while their primary context involved comorbid conditions (e.g., post-stroke AF, cardiac surgery populations), they assessed anxiety using validated instruments in adult AF patients and reported AF-relevant outcomes meeting our PICO criteria. The rationale for inclusion of each borderline study is documented in the supplementary data extraction table.

### Data extraction and synthesis

Data was extracted independently by two authors using Rayyan QCRI for the following: author, publication year, country, study design (cohort, case-control, cross-sectional, or randomized controlled trial), total sample size, age and sex demographics of participants, type of anxiety assessment (e.g., GAD-7, HADS, Beck Anxiety Inventory), method of administration, and diagnostic threshold; characteristics of atrial fibrillation: form (paroxysmal, persistent, or permanent), diagnostic criteria, and methods of confirmation. Comparison groups included patients without anxiety, patients with anxiety but no AFib, and the general population. Primary outcomes include AFib incidence and recurrence rate, symptom burden (e.g., palpitations, fatigue, chest discomfort), rate of hospitalization, treatment outcomes (e.g., response to antiarrhythmics, ablation success), quality of life and mortality, range, and mean duration of follow-up.

A qualitative (narrative) synthesis was used in preference to meta-analysis. Although a subset of studies employed common anxiety instruments (GAD-7, HADS, ZSAS), formal pooling was considered inappropriate due to substantial clinical and methodological heterogeneity across included studies: study designs ranged from large population-based cohorts to small single-centre RCTs; anxiety was operationalised as a continuous score, a binary threshold, or a clinical diagnosis; AF outcomes included incidence, recurrence, symptom burden, and quality of life; and follow-up periods varied from weeks to several years. These differences preclude meaningful statistical pooling without risking misleading summary estimates. Key findings were therefore synthesised under thematic domains: anxiety as a risk factor for AF incidence and recurrence; mechanisms linking anxiety and AF; impact of anxiety on symptom burden and healthcare utilisation; and the effect of interventions on disease burden.

### Risk of bias assessment

**Figure 2.**
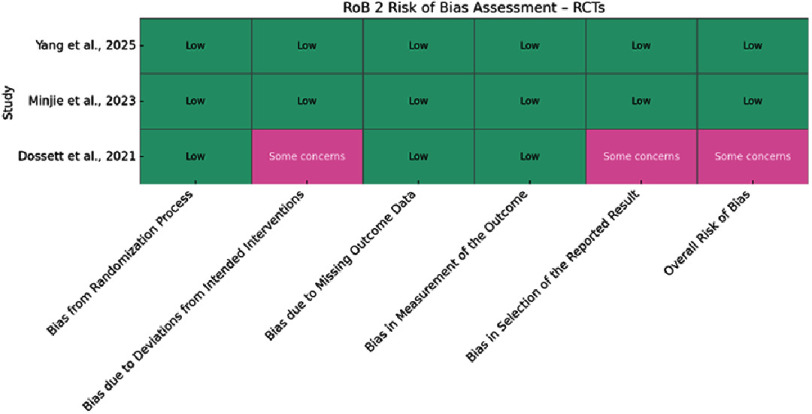
Cochrane risk of bias table.

Of the 84 included studies (see Table 1), most were observational and were assessed for methodological quality using the Newcastle-Ottawa Scale (NOS). Table 2 presents the full NOS assessments for the eight representative observational studies selected to illustrate the range of methodological quality across the corpus; a complete NOS table covering all included observational studies is provided as a Supplementary File. Across all assessed observational studies, 23 (33%) demonstrated low risk of bias, 44 (63%) moderate risk, and 3 (4%) high risk, with the main sources of concern being small sample sizes and insufficient adjustment for confounding variables. The RCTs included in the review were assessed using the Cochrane Risk of Bias 2 (RoB 2) tool (Figure 2). These studies generally showed low risk of bias across the five RoB 2 domains (randomisation process, deviations from intended interventions, missing outcome data, measurement of the outcome, and selection of reported results), with the exception of four studies that raised some concerns, primarily regarding incomplete follow-up and absence of blinded outcome assessment. Overall, the body of evidence was of acceptable quality to support a narrative synthesis, though the observational design of the majority of included studies limits causal inference.

**Table 1:** Full results table. The number in the first column relates to the reference number in the references.

**No.**	**Study type**	**Population**	**Intervention/ Exposure**	**Comparison**	**Outcome**	**Key finding**
^1^	RCT	68 AF patients	Family-focused intervention	Standard care	AFEQT, ICE-FPSQ, anxiety, depression	Improved QoL and caregiver experience; no significant anxiety or depression difference
^2^	RCT	AF patients	Structured education on AF	Standard care	QoL, AF knowledge, adherence, anxiety/depression	Significant improvements in QoL, knowledge, and adherence; no significant changes in anxiety or depression
^3^	RCT	338 patients	AF ablation (PVI ± PWI)	Men vs. women	AFEQT, HADS, arrhythmia recurrence	Women had higher baseline anxiety; similar improvements post-ablation; anxiety not predictive of recurrence
^4^	Mendelian randomization	Genetic cohort	Genetic predisposition to anxiety/ depression/ panic	Genetically non-predisposed	Risk of AF	Anxiety not causally related to AF; depression and panic are; depression partially mediated by HTN & obesity
^5^	Cross-sectional	278 chronic/AF patients	Sociodemographic & clinical characteristics	N/A	Understanding, care satisfaction	Majority older men; high satisfaction; no direct AF-anxiety outcome
^6^	Cross-sectional	Chronic illness patients	Patient self-report	None	Demographics, hospitalizati on, care perception	Mostly elderly, pensioners; 51.6% felt well-informed
^7^	Cross-sectional survey	116 AF patients	Anxiety, depression, frailty	None	QoL (ASTA III)	Higher anxiety, depression, frailty → worse QoL
^8^	Observational	Patients monitored for AF	Trait anxiety, age, comorbidity	No AF vs. AF	Risk of AF	Trait anxiety, older age, comorbidity → AF risk
^9^	Case-control	720 participants	ZSAS, CES-D	Controls without AF	Anxiety, depression, comorbidities	AF group had higher anxiety; anxiety & CHD/stroke linked to AF
^10^	Observational	Caregivers of AF patients	Caregiver/ patient anxiety/ depression	None	Caregiver mental health	Caregiver depression linked to own comorbidities & patient depression; anxiety linked to age, sex, patient anxiety
^11^	Observational	110 participants	Smartwatch prescription	Smartwatch users vs. non-users	Anxiety, activation, self-rated health, AF detection	No significant change in anxiety, activation, or health; AF incidence 6% in smartwatch users
^12^	Interventional	52 AF patients	Shared Decision Making (SDM)	Pre-SDM vs. Post-SDM	Anxiety, OAC decision-making	Anxiety decreased post-SDM; OAC uptake increased; anxiety associated with gender
^13^	Cross-sectional + regression	164 AF patients	Income, disease course, episode frequency	N/A	Anxiety, depression	Anxiety (34.15%) and depression (25.61%) linked to income, AF course, episode duration; age linked to depression
^14^	Retrospective cohort	AF risk group	Medication use, age, gender	Varying exposures	Incident AF and AF burden	Digoxin + AF risk; SSRIs, SNRIs, Sotalol + AF burden; older age + AF risk; male gender + AF risk
^15^	Observational	171 participants	Mood/anxiety disorders, HF, digoxin use	With vs. without symptoms	AF symptom severity, QoL, depression/anxiety	Mood/anxiety disorders linked to AF symptoms and QoL; HF and digoxin linked to depressive symptoms
^16^	RCT	AF patients	Cognitive Behavioral Therapy (CBT)	CBT vs. TAU	Anxiety, depression, QoL, illness perception	CBT improved anxiety, illness perception, mental QoL; no difference in physical QoL and PHQ-9
^17^	Retrospective matched cohort	484 HF patients	Cardiac Rehabilitation (CR)	HF + AF vs. HF only	ISWT, HRQOL, HADS	Both groups improved post-CR; AF group started with lower exercise capacity; final outcomes similar
^18^	Quantitative	AF patients	Cognitive-behavioral and illness representation cluster analysis	Cluster 1 vs. Cluster 2	QoL, HADS	Negative illness/ emotional representations → worse QoL and more symptoms
^19^	Longitudinal	Post-stroke patients	Time since stroke	Baseline values	EQ-5D-3L, HADS	HRQOL improved over 12 months; anxiety reduced; AF had small but significant effect
^20^	Observational	295 patients undergoing CBA	Elevated vs. low HADS score	HADS groups	QoL (pre and post CBA)	All improved post-CBA; higher HADS scores → lower QoL before and after
^21^	Qualitative	AF patients	Living with AF and care system	N/A	Thematic analysis	Challenges in understanding, managing symptoms, adapting identity
^22^	Observational	175 AF patients	Symptom cluster analysis	Cluster group 1 vs. group 2	Psychological distress, QoL	More symptoms → higher anxiety/ depression, lower QoL
^23^	Prospective cohort	2,615 participants	Irregular HR alarm *via* Apple Watch/BP cuff	Alarm responders vs. general	Anxiety, AF diagnosis, false positives	Mild anxiety in 34.2%; 6.6% diagnosed with AF; most alarms false positives
^24^	RCT	Cardiovascular patients	EECP therapy	EECP vs. control	BP, LVEF, LAD, anxiety, depression	EECP improved cardiac function and reduced anxiety/depression
^25^	Observational	AF patients	Catheter ablation	Pre vs. post CA	AFQLQ, BNP, LVEF, LAD	Significant improvement in symptoms and cardiac function
^26^	Cross-sectional survey	1,244 AF patients	Frailty, depression, anxiety, social factors	Married vs. unmarried; educated vs. uneducated	AFQOL	Poor AFQOL linked to frailty, mental health, isolation; marriage and education protective
^27^	Retrospective cohort	205,019 AF patients	Mental health conditions, OAC therapy	No MHCs	Bleeding risk	MHCs linked to higher bleeding risk; OAC therapy increased bleeding risk
^28^	RCT (pilot)	Post-cardiac surgery patients	Acupuncture	Standard care	Post-op AF, anxiety, ICU stay	Acupuncture reduced AF, anxiety, ICU stay duration
^29^	Cross-sectional survey	531 AF patients	Personality types	Other personality types	QoL, GAD-7, PHQ-9	Sanguine personality → better QoL, lower anxiety/depression
^30^	Comparative cohort	80 patients	Nursing intervention	Control group	Cardiac function, hospital stay, emotional wellbeing	Nursing improved LVEF, reduced complications, enhanced well-being
^31^	Cohort	203,154 AF patients	Mental health conditions	No MHC	Stroke, mortality, OAC use	MHCs → higher crude stroke and mortality; OAC use reduced risks
^32^	Population-based cohort	146,377 AF patients	AF diagnosis	Matched controls	Psychiatric disorders, medication use	AF patients had higher psychiatric diagnoses and prescriptions
^33^	Cross-sectional	104 patients with AHI ≥ 15	OSA severity (Baveno groups)	Groups A-D	Depression, anxiety, HADS	Higher OSA severity → higher depression; anxiety not significantly different
^34^	Observational	AF patients	Catheter ablation	Pre vs. post	AF burden, QoL	AF burden dropped to 0%; QoL improved
^35^	Survey	1320 AF patients	Ablation outcomes	N/A	Readmissions	36% acute, 43% planned readmissions; linked to IHD, anxiety, depression
^36^	Cross-sectional	300 AF patients	TEE experience	Inpatient vs. outpatient	Anxiety, discomfort, hospital stay	Outpatient TEE → shorter LOS, lower costs; high discomfort overall
^37^	Case-control	AF patients	Dynamic ECG and psychiatric factors	Observation vs. control	Arrhythmia detection, psychological factors	Anxiety/depression linked to age, education, OCD, somatization, alcohol
^38^	Cohort	6.6 million people	Mental disorders	No mental disorders	AF incidence	Mental disorders → higher AF risk; bipolar/schizophrenia: 2x risk; anxiety/depression: 1.5–1.7x risk
^39^	Mediation study	AF patients	AF knowledge, coping styles	N/A	Anxiety, depression, life satisfaction	Coping mediates between knowledge and psychological outcomes
^40^	Prospective	346 AF patients	Catheter ablation	Pre vs. post ablation	AF burden, AFEQT score	AF burden dropped to 0%; burden reduction improved QoL
^41^	Real-world cohort	2,769 AF patients	ARENA intervention + OAC use	Intervention vs. control	OAC adherence, re-hospitalization, QoL, anxiety	Higher adherence, lower re-hospitalization; anxiety reduced
^42^	Comparative cohort	80 patients	Nursing intervention	Control group	Cardiac function, hospital stay, emotional wellbeing	Improved LVEF, reduced complications, enhanced satisfaction
^43^	Pre-post & waitlist control	18 participants	AF program	Waitlist control	QoL, depression, anxiety, AF symptoms	QoL and depression improved; anxiety not significantly improved
^44^	Cross-sectional	950 AF patients	Demographics & clinical factors	N/A	AF knowledge level	Older age, lower education, anxiety linked to lower AF knowledge
^45^	Regression & mediation	178 AF patients (Beijing, China)	Illness perception, GAD, coping strategies	N/A	HRQOL scores	GAD and coping mediated illness perception’s impact on QoL
^46^	Matched cohort	42,038 patients	AF ablation	No ablation	Mental health, dementia, stroke	Ablation reduced anxiety, depression, suicidality, dementia, stroke
^47^	Comparative management	Unknown	Warfarin education & management	Control group	Knowledge, compliance, anxiety, depression	Education improved compliance, satisfaction, reduced anxiety/depression
^48^	Longitudinal	Unknown	Surgery	Pre vs. post	GAD and depression scores	Scores reduced post-surgery; prevalence unchanged; linked to age, stroke
^49^	Population cohort	2.5M diabetic patients	Mental disorders	No mental disorders	Risk of AF	Anxiety, insomnia, depression increased AF risk
^50^	Observational	147 ICD patients	Age ≥60, absence of AF, female gender	Younger, AF present, male	QoL, anxiety, ICD acceptance	Older age, female gender, absence of AF predicted better outcomes
^51^	Prospective cohort	General population	Healthy sleep pattern	Poor sleep pattern	Depression, anxiety	Healthy sleep reduced depression and possibly anxiety
^52^	Observational	93 participants	High perceived efficacy in physician interaction	Low efficacy	Mental/physical health, anxiety	High efficacy → better mental health and activation; no link to anxiety
^53^	Comparative analysis	Smartwatch users	Smartwatch tech use	Non-users	Anxiety, activation, health	No major differences; smartwatch users had worse mental health perception
^54^	Observational	142 stroke patients	Recovery indices	Improved vs. unimproved	Recovery scores, anxiety/depression	Anxiety/depression prominent; plaque index cutoff significant
^55^	Registry study (FinACAF)	239,222 AF patients	Mental health condition	No MHC	OAC initiation	MHCs linked to lower OAC initiation; trend persisted in NOAC era
^56^	RCT	96 participants	Prior smartwatch ownership	No prior smartwatch	Physical/mental health, anxiety	Owners reported better physical health; no difference in anxiety
^57^	Cross-sectional observational	NVAF patients on oral anticoagulants (Spain)	Oral anticoagulant type (DOAC vs VKA)	DOACs vs. VKAs	HRQL (EQ-5D-5L)	DOAC group reported slightly better QoL than VKA group, mainly due to lower anxiety/depression scores; no significant difference in other EQ-5D-5L dimensions
^58^	Observational cohort	2431 patients	Insomnia and comorbidities	Non-insomnia group	Depression, anxiety, AF, dementia	Insomnia linked to higher depression, anxiety, AF
^59^	Retrospective/prospective	182 PVI patients	Ablation method	Group comparisons	Anxiety, pain, discharge rate	vHPSD shortest ablation time; no group differences in anxiety
^60^	Prospective cohort	650 stroke/TIA patients	Stroke severity, diabetes, AF	Mild vs. moderate/ severe	Self-reported health status	NIHSS and AF predicted more severe impairment
^61^	Longitudinal survey	129 AF patients	Time since AF diagnosis	Newly vs. previously diagnosed	Symptom burden, HRQOL	Newly diagnosed had better outcomes at 6 months
^62^	Retrospective analysis	385 acute HF patients	Comorbidities and clinical factors	N/A	Mortality	Haemodialysis and depression/ anxiety were significant mortality risks
^63^	Cross-sectional	95 participants	Low-income status	High vs. low income	Anxiety, physical health	Low income → higher anxiety, lower physical health
^64^	Qualitative	Not specified	Patient experiences with arrhythmia	N/A	Self-management themes	Identified themes: managing anxiety, self-efficacy, communication
^65^	Mixed-methods	87 + 104 participants	SDM materials for dabigatran	Before vs. after viewing	Anxiety, confidence in HCPs	SDM reduced anxiety, increased confidence
^66^	Qualitative interviews	Patients & HCPs	Risk prediction models	N/A	Attitudes, barriers	Supportive of models; emphasized education and protocols
^67^	Prospective	555 older adults	Anxiety & depression scores	Adjusted for CV risk	Coronary events, mortality	Anxiety/depression increased coronary event risk independently
^68^	Survey	401 AF patients	Demographics and comorbidities	N/A	QoL, anxiety, depression	34% depressed, 27% anxious; poorer scores linked to TIA, diabetes
^69^	Retrospective database	2,096 pericarditis patients	Recurrence history	N/A	Complications, comorbidities	High anxiety (21%) and depression (14%) rates
^70^	Retrospective cohort	Patients over 5 years	Antiplatelet therapy persistence	Persistent vs. non-persistent	Medication adherence	AF, anxiety, female sex linked to non-persistence
^71^	Retrospective cohort	Periodontitis patients	Periodontitis	Non-periodontitis	Stroke incidence	Anxiety increased stroke risk; AF, HTN also risk factors
^72^	Observational	24,017 BC survivors	Breast cancer survivorship	Matched controls	HF, AF, anxiety/ depression	BC survivors had higher risks for HF, AF, anxiety/depression
^73^	Observational (pre-post)	821 women with palpitations	Smartphone ECG + tracking	Pre-intervention	ECG findings, anxiety, HRQOL	94% benign ECG; decreased anxiety/depression post-intervention
^74^	Survey	116 AF patients	Educational videos on AF	Self-report pre/post	Satisfaction, anxiety, adherence	≥98% satisfied; reduced anxiety, improved decision-making
^75^	Registry study	2,454 stroke patients	Comorbidity burden (CCI ≥2)	CCI = 0	Mortality, disability	Multimorbidity → increased mortality and disability
^76^	Validation study	Stroke registry data	ICD-10-AM vs. pharmacy data	Clinical trial data	Comorbidity detection	Pharmacy data better for anxiety detection
^77^	Mendelian Randomization	>3 million patients	Genetic risk for CHD/MHD	None	Causal relationships	CHD increases MDD/ mania; HF increases bipolar/schizophrenia risk
^78^	Observational	Medication adherence group	Regular intake	Comparison group	Adherence, comorbidities	Regular intake associated with AF, stroke, DM
^79^	Registry (Türkiye)	2.72 million HF patients	Comorbidity profiles	Age/sex/SES groups	Comorbidity prevalence	Anxiety in 48.1%; ≥5 comorbidities more common with age
^80^	Retrospective cohort	551,586 COVID-19 patients	COVID-19 infection	Non-COVID cohort	Post-COVID complications	Increased risk for AF, anxiety, HF, mortality
^81^	Registry (FinACAF)	67,503 AF patients on DOACs	Mental health conditions	No MHC	DOAC nonpersistence	MHCs increased DOAC discontinuation; anxiety not significant
^82^	RCT	100 AF patients	Catheter ablation	Medical therapy	Psychological distress, AF burden	Ablation reduced distress, anxiety, depression, AF burden
^83^	Observational	1353 AF patients	Generalized and cardiac anxiety	N/A	HRQOL	Higher generalized anxiety → reduced HRQOL
^84^	Systematic review	2591 participants	Psychological interventions	No intervention	Depression and anxiety	Moderate reduction in depression and anxiety symptoms

**Table 2:** Newcastle–Ottawa Scale (NOS)–Observational Studies.

Study (Author, Year)	Selection (0–4)	Comparability (0–2)	Outcome/ Exposure (0–3)	Total score (out of 9)	Bias level
Shen et al., 2021	3	1	2	6	Moderate
Bang & Park, 2023	4	2	3	9	Low
Yan et al., 2024	4	2	2	8	Low
Lomper et al., 2023	3	1	2	6	Moderate
Meng et al., 2024	3	2	2	7	Low
Yu et al., 2022	3	1	2	6	Moderate
Ahn et al., 2022	4	2	3	9	Low
Pierre-Louis et al., 2023	4	2	3	9	Low

## Results

A total of 1,804 records were identified through database searches. After removal of duplicates, 1,661 records were screened by title and abstract, of which 100 full-text articles were assessed for eligibility. Of these, 16 were excluded at full-text stage for the following reasons: population did not meet eligibility criteria (*n* = 6; including studies focused exclusively on paediatric populations or non-cardiac primary outcomes); outcomes did not include a validated anxiety measure or AF-relevant endpoint (*n* = 5); study design not eligible (*n* = 3; protocols and conference abstracts without full data); and duplicate publication reporting the same dataset (*n* = 2). Eighty-four studies met the inclusion criteria and were included in the final synthesis. The included studies were published between 2020 and 2025 and represented diverse geographic regions. Sample sizes varied widely, ranging from small clinical cohorts to large population-based studies, with participants predominantly adults diagnosed with paroxysmal, persistent, or permanent atrial fibrillation. Anxiety was assessed using validated instruments such as the Hospital Anxiety and Depression Scale (HADS), Generalized Anxiety Disorder-7 (GAD-7), Zung Self-Rating Anxiety Scale (ZSAS), and Atrial Fibrillation Effect on Quality-of-Life (AFEQT/ASTA) questionnaires.

Across the included studies, anxiety was highly prevalent among patients with atrial fibrillation, with reported rates consistently exceeding those observed in the general population. Several observational studies demonstrated that anxiety was independently associated with an increased risk of atrial fibrillation onset, particularly among younger individuals and those without significant structural heart disease. Anxiety was also identified as a predictor of atrial fibrillation recurrence following cardioversion or catheter ablation, with anxious patients experiencing higher symptom burden and more frequent arrhythmic episodes during follow-up.

Higher anxiety levels were consistently associated with worse symptom perception and impaired health-related quality of life. Patients with elevated anxiety scores reported more frequent palpitations, dyspnea, fatigue, and exercise intolerance, despite similar objective measures of arrhythmia severity. Anxiety was also linked to increased healthcare utilization, including emergency department visits and hospitalizations, suggesting an amplification of symptom awareness and distress in affected individuals.

Anxiety adversely influenced treatment outcomes in atrial fibrillation. Several studies reported lower adherence to oral anticoagulation and antiarrhythmic medications among anxious patients. Anxiety was associated with poorer outcomes following catheter ablation, including higher recurrence rates and reduced patient satisfaction. Conversely, interventions such as successful rhythm control, structured patient education, shared decision-making, and psychosocial support were associated with significant reductions in anxiety levels and improvements in quality of life.

Risk of bias assessment revealed that the majority of included studies were of low methodological risk, with approximately two-thirds demonstrating adequate selection, comparability, and outcome assessment. A smaller proportion of studies showed moderate risk of bias, primarily due to limited adjustment for confounding variables or short follow-up duration, while only a minority were judged to be at high risk of bias. Overall, the evidence consistently supported a clinically relevant association between anxiety and adverse atrial fibrillation-related outcomes.

## Discussion

Atrial fibrillation (AF) is one of the most frequently observed arrythmia. It is clinically linked to a number of other diseases, mainly thromboembolic stroke and heart failure. The prevalence of AF is drastically rising because of several factors such as aging population and lifestyle changes^5^. It also leads to a diminished health related quality of life (HRQoL)^18^. A cross sectional analysis of a prospective cohort study performed on 1244 participants aged 65+ revealed that 42% reported to have poor AF quality of life (AFQoL). Many factors are associated with having poor AFQoL such as anxiety, depression, fraility and social isolation^29^.

Previous studies have shown that mental illnesses are associated with cardiovascular diseases (CVD)^5,33^. A study carried out on 150 participants concluded that among the elderly patients with CVD, anxiety and depression are associated with occurrence of arrhythmias^40^. In addition, a study was conducted in Denmark on 146,377 AF patients and 292,754 controls showed that incident AF patients were at higher risk of having psychiatric outpatients or hospital contacts and were also more likely to fill prescription for psychotropic drugs as compared to the healthy population during the first 3 months after diagnosis of AF^35^.

Mental health conditions (MHCs) such as anxiety and depression, may increase the chance of developing AF^5^. Among AF patients, anxiety and depression are highly prevalent, with multiple included studies reporting rates of 30–40% depending on the assessment tool and population studied. Furthermore, a study done on 2,473,005 CHD and 803,801 MHD patients found a positive correlation between AF and Generalized Anxiety Disorder (GAD).

A bidirectional relationship is observed between anxiety and AF^16^. Anxiety causes an increase in the frequency of symptoms which leads to an increase in the severity of symptom experience^7,24^. On the other hand, patients can be excessively concerned about their AF symptoms which can result in the development of anxiety^18^.

Numerous studies have demonstrated a correlation between AF and anxiety. A case control study conducted on 720 participants in China revealed that a positive association existed between anxiety and AF after adjusting for a history of CHD, valvular heart disease, hypertension, stroke, hyperlipidemia, and diabetes, as well as depression score^9^. Another study carried out on 6,576,582 young adult subjects demonstrated that young adults with depression, insomnia and anxiety disorder had a 1.5 to 1.7-fold higher risk of incident AF than those without these MHCs^41^. Even among diabetic patients, MHCs such as depression, insomnia and anxiety are linked with an increased likelihood of AF, as shown in a study done on 2,512,690 patients^53^.

The level of anxiety experienced by AF patients depends on a variety of factors. These are the duration since AF diagnosis, frequency and length of AF episodes during the past month. Additionally, it also depends on the per capita monthly household income^15^.

Apart from affecting the HRQoL as mentioned earlier, anxiety is also associated with an increased likelihood of AF occurrence after treatment procedures such as cardioversion or circumferential pulmonary vein ablation. Furthermore, it is also linked with an elevated risk of ischemic stroke and intracranial hemorrhage in AF patients starting warfarin therapy. This may be the result of psychological, physiological (inflammation, sympathetic hyperactivity), and behavioural factors including reduced treatment adherence^15,53^.

Although the exact mechanism by which AF and anxiety interact is not completely understood, several pathophysiological pathways have been proposed in the background literature. Cardiac autonomic dysfunction—characterised by sympathetic hyperactivity and parasympathetic withdrawal—is considered a leading candidate mechanism^5,9,15^. During anxiety states, overactivation of the sympathetic nervous system results in increased catecholamine release, which may promote atrial ectopy and AF^15,53^. Furthermore, sympathetic hyperactivity stimulates the renin-angiotensin-aldosterone system, leading to elevated angiotensin II levels that promote atrial fibrosis, a substrate for AF development and maintenance^53^. Inflammatory pathways represent a further proposed mechanism: elevated levels of C-reactive protein, IL-6, and TNF-*α* during psychological stress may contribute to atrial electrical and structural remodelling^15,53^.

It should be noted that these mechanistic pathways are largely drawn from background literature and pre-clinical evidence; the studies systematically reviewed here were primarily observational and did not directly measure these biological mediators, so causal inferences about mechanisms must be made with caution. The most methodologically robust finding in this review with respect to causality comes from one included Mendelian randomisation (MR) study^5^, which used genetic instrumental variables to interrogate causal relationships between common mental health conditions and AF. Crucially, this study found no evidence of a causal effect of anxiety on AF incidence. By contrast, depression and panic disorder showed statistically significant causal associations with AF in the MR framework. This finding materially qualifies the conclusions that can be drawn from the broader observational evidence base: the consistent observational association between anxiety and AF-related outcomes observed across other included studies may reflect shared risk factors, reverse causation, or residual confounding rather than a direct causal pathway from anxiety to AF. The implications of this MR evidence are important for clinical translation: while addressing anxiety remains warranted for patient wellbeing and quality of life, the available evidence does not support anxiety as an independent causal driver of AF onset. Future research should examine whether the observed associations are mediated by depression or panic symptoms co-occurring with anxiety, and whether interventions targeting these conditions specifically reduce AF burden.

As per the present guidelines, antiarrhythmic drugs and catheter ablation can be used to alleviate symptoms of AF^18^. Several treatment methods have resulted in decreased anxiety as well. According to a retrospective cohort study, catheter ablation in AF patients might lead to a lower chance of developing various MHCs such as anxiety, depression, insomnia, suicidal ideation or attempt, and dementia, as compared to those who did not undergo this procedure^50^. Furthermore, compared to medical therapy, AF patients who underwent catheter ablation have been shown to experience improvements in psychological distress, consistent with the broader finding that ablation reduces psychiatric disorder risk in this population^50^. Moreover, the resultant reduction in AF burden after catheter ablation is associated with improvement in the quality of life^43^. Beyond procedural interventions, shared decision-making approaches to anticoagulation have been shown to reduce anxiety and improve treatment adherence in AF patients, underscoring the value of patient-centred communication in this condition^13^.

Another treatment option is Enhanced External Counterpulsation (EECP), which reduces myocardial ischemia. An RCT conducted in China using 100 patients diagnosed with paroxysmal AF (PAF) demonstrated that the level of anxiety and depression was low among both groups (EECP and pharmacological therapy). However, those who underwent EECP had significantly lower anxiety and depression scores compared to the group who received pharmacological therapy (β-blockers or propafenone)^26^.

This indicates that physicians should include an assessment of psychiatric risk factors along with the evaluation of the clinical indicators^15,50^. This will help the clinicians in choosing the best treatment option, thus improving patient outcomes and quality of life^29^.

However, there are some limitations of this review. The included studies differ in study design, population size, and the outcome measured which restricts the generalizability of the findings. Furthermore, the differences in the diagnostic criteria and measurement of AF and anxiety disorders across the studies could result in inconsistency.

Additional longitudinal studies should be performed on diverse populations using standardized definitions and tools in order to corroborate the current findings.

## Conclusion

This systematic review of 84 studies provides evidence of a clinically significant association between anxiety and atrial fibrillation, though the nature of this relationship is complex and not straightforwardly causal. The majority of included observational studies consistently demonstrated that anxiety is prevalent among AF patients and associated with greater symptom burden, impaired health-related quality of life, reduced treatment adherence, and worse outcomes following ablation or cardioversion. These findings support the routine incorporation of psychological assessment into AF care pathways. However, the Mendelian randomisation evidence identified in this review found no causal effect of anxiety on AF incidence—in contrast to depression and panic disorder—which requires that the observational associations be interpreted cautiously. Addressing anxiety in AF patients remains important for reducing symptom distress and improving quality of life, but clinicians should be aware that treating anxiety may not directly reduce AF recurrence or burden.

Among the interventions discussed in this review, catheter ablation was associated with a reduced risk of developing psychiatric disorders—including anxiety, depression, and insomnia—compared with patients managed without ablation^50^, and was also linked to improvements in health-related quality of life through reduction of AF burden^43^. Pre-operative anxiety assessment is warranted given its association with post-ablation AF recurrence. Structured patient education and shared decision-making approaches were additionally associated with reduced anxiety and improved adherence in included studies^13^. Integrated management of anxiety in AF patients therefore has the dual benefit of addressing patient wellbeing and potentially improving engagement with treatment, even if a direct causal effect on AF arrhythmogenesis remains to be established. Future longitudinal studies using standardised anxiety instruments and clearly defined AF outcomes are needed to resolve outstanding questions about causality and to guide more comprehensive, patient-centred care.

## References

[1] Rosenstrøm S, Risom SS, Kallemose T, Dixen U, Hove JD, Brødsgaard A (2023). Clinical outcomes of a short-term family-focused intervention for patients with atrial fibrillation-A randomised clinical trial. PloS one.

[2] Li PWC, Yu DSF, Yan BP (2025). Nurse-led multicomponent behavioral activation intervention for patients with atrial fibrillation: A randomized controlled trial. Circulation. Arrhythmia and electrophysiology.

[3] Segan L, Chieng D, Crowley R, William J, Sugumar H, Ling LH, Hawson J, Prabhu S, Voskoboinik A, Morton JB, Lee G, Sterns LD, Ginks M, Sanders P, Kalman JM, Kistler PM (2024). Sex-specific outcomes after catheter ablation for persistent AF. Heart rhythm.

[4] Pogosova NV, Badtieva VA, Ovchinnikova AI, Sokolova OY, Vorobyeva NM (2022). Efficacy of secondary prevention and rehabilitation programs with distant support in patients with atrial fibrillation after intervention procedures: impact on psychological status. Kardiologiia.

[5] Zhou H, Ji Y, Sun L, Wang Z, Jin S, Wang S, Yang C, Yin D, Li J (2024). Exploring the causal relationships and mediating factors between depression, anxiety, panic, and atrial fibrillation: a multivariable Mendelian randomization study. Journal of affective disorders.

[6] Polikandrioti M (2021). Atrial fibrillation: the impact of anxiety and depression on patients’ needs. Psychiatrike Psychiatriki.

[7] Lomper K, Ross C, Uchmanowicz I (2023). Anxiety and depressive symptoms, frailty and quality of life in atrial fibrillation. International journal of environmental research and public health.

[8] Alkan Kayhan S, Güner E, Hanedan MO, Topal Çolak E, Mataraci I (2022). Relationship between preoperative anxiety and atrial fibrillation after coronary artery bypass graft surgery. The journal of nursing research: JNR.

[9] Shen ZX, Sun YM, Gu HH, Zhang Y, Shen ZW, Liang XN, Ding D, Wang J (2021). Association between anxiety symptoms and atrial fibrillation in a community cohort of Chinese older adults: a case-control study. BMC cardiovascular disorders.

[10] Li S, Li Q, Jiang C, Chen X, Lai Y, He L, Cui F, Wu J, Hu R, Jia C, Feng L, Sang C, Tang R, Long D, Du X, Dong J, Ma C (2022). Factors associated with depression and anxiety among caregivers of patients with atrial fibrillation. Journal of clinical nursing.

[11] Oser M, Khan A, Kolodziej M, Gruner G, Barsky AJ, Epstein L (2021). Mindfulness and interoceptive exposure therapy for anxiety sensitivity in atrial fibrillation: a pilot study. Behavior modification.

[12] Paul TJ, Tran KV, Mehawej J, Lessard D, Ding E, Filippaios A, Howard-Wilson S, Otabil EM, Noorishirazi K, Naeem S, Hamel A, Han D, Chon KH, Barton B, Saczynski J, McManus D (2023). Anxiety, patient activation, and quality of life among stroke survivors prescribed smartwatches for atrial fibrillation monitoring. Cardiovascular digital health journal.

[13] Chiu HH, Chang SL, Cheng HM, Chao TF, Lin YJ, Lo LW, Hu YF, Chung FP, Liao JN, Tuan TC, Lin CY, Chang TY, Kuo L, Liu CM, Tsai YN, Huang YT, Chang YL, Wung JC, Chen SA (2023). Shared decision making for anticoagulation reduces anxiety and improves adherence in patients with atrial fibrillation. BMC medical informatics and decision making.

[14] Guo XY, Wang Z, Li ST, Jiang C, Sang CH, Ma CS (2023). Analysis of factors associated with anxiety in patients with atrial fibrillation and their caregivers. Zhonghua yi xue za zhi.

[15] Qian L, Shen Y (2024). Anxiety and depression in patients undergoing catheter ablation due to atrial fibrillation: a cross-sectional survey. Heliyon.

[16] Koh Y, Kwok C, Voskoboinik A, Kalman JM, Wong M (2023). Serotonin antidepressants and atrial fibrillation burden from cardiac implantable electronic devices. Journal of arrhythmia.

[17] Koleck TA, Mitha SA, Biviano A, Caceres BA, Corwin EJ, Goldenthal I, Creber RM, Turchioe MR, Hickey KT, Bakken S (2021). Exploring depressive symptoms and anxiety among patients with atrial fibrillation and/or flutter at the time of cardioversion or ablation. The Journal of cardiovascular nursing.

[18] Minjie Z, Zhijuan X, Xinxin S, Xinzhu B, Shan Q (2023). The effects of cognitive behavioral therapy on health-related quality of life, anxiety, depression, illness perception, and in atrial fibrillation patients: a six-month longitudinal study. BMC psychology.

[19] Alhotye M, Evans R, Ng A, Singh SJ (2023). Cardiac rehabilitation for heart failure and atrial fibrillation: a propensity- matched study. Open heart.

[20] Taylor EC, O’Neill M, Hughes LD, Moss-Morris R (2022). Atrial fibrillation, quality of life and distress: a cluster analysis of cognitive and behavioural responses. Quality of life research: an international journal of quality of life aspects of treatment, care and rehabilitation.

[21] Sadlonova M, Wasser K, Nagel J, Weber-Krüger M, Gröschel S, Uphaus T, Liman J, Hamann GF, Kermer P, Gröschel K, Herrmann-Lingen C, Wachter R (2021). Health-related quality of life, anxiety and depression up to 12 months post-stroke: influence of sex, age, stroke severity and atrial fibrillation - A longitudinal subanalysis of the Find-AF(RANDOMISED) trial. Journal of psychosomatic research.

[22] Raileanu G, Jawid N, Bohte E, Hof IE, Khan M, de Ruiter GS, Verbeek EC, de Jong JSSG, Mol D (2024). Do depressive and anxiety symptoms influence the quality of life of patients with atrial fibrillation after cryoballoon ablation: a comparison study. Journal of interventional cardiac electrophysiology: an international journal of arrhythmias and pacing.

[23] Holmlund L, Hellström Ängerud K, Hörnsten Å, Valham F, Olsson K (2023). Experiences of living with symptomatic atrial fibrillation. Nursing open.

[24] Bang C, Park S (2023). Symptom clusters, psychological distress, and quality of life in patients with atrial fibrillation. Healthcare (Basel, Switzerland).

[25] Pastapur A, Pescatore NA, Shah N, Kheterpal S, Nallamothu BK, Golbus JR (2023). Evaluation of atrial fibrillation using wearable device signals and home blood pressure data in the michigan predictive activity & clinical trajectories in health (MIPACT) study: a subgroup analysis (MIPACT-AFib). Frontiers in cardiovascular medicine.

[26] Yang F, Zhang Y, Jiang Y, Wei Q, Cheng X, Xiao J, Chen G (2025). The effect of enhanced external counterpulsation on anxiety and depression in patients with paroxysmal atrial fibrillation: a randomized controlled trial. Frontiers in cardiovascular medicine.

[27] Gémes K, Malmo V, Str LB, Ellekjær H, Loennechen JP, Janszky I, Laugs LE (2024). Insomnia symptoms and risk for atrial fibrillation - The HUNT study. Journal of sleep research.

[28] Kolba NK, Lee B, Tannous HJ, Bilfinger TV, Shroyer AL (2023). Preoperative mental illness and postoperative atrial fibrillation in cardiac surgery patients: identifying a vulnerable population. Journal of clinical and translational science.

[29] Pierre-Louis IC, Saczynski JS, Lopez-Pintado S, Waring ME, Abu HO, Goldberg RJ, Kiefe CI, Helm R, McManus DD, Bamgbade BA (2023). Characteristics associated with poor atrial fibrillation-related quality of life in adults with atrial fibrillation. Journal of cardiovascular medicine (Hagerstown, Md.).

[30] Teppo K, Jaakkola J, Biancari F, Halminen O, Linna M, Putaala J, Mustonen P, Kinnunen J, Jolkkonen S, Niemi M, Hartikainen J, Airaksinen KEJ, Lehto M (2022). Mental health conditions and bleeding events in patients with incident atrial fibrillation: a finnish nationwide cohort study. General hospital psychiatry.

[31] Feingold KL, Moskowitz JT, Elenbaas C, Andrei AC, Victorson D, Kruse J, Grote V, Patil KD, Shafiro T, Grimone A, Lin F, Davidson CJ, Ring M, McCarthy PM (2023). Acupuncture after valve surgery is feasible and shows promise in reducing postoperative atrial fibrillation: The ACU-Heart pilot trial. JTCVS open.

[32] Yan Q, Liang J, Yuan Y, Li Y, Fan J, Wu W, Xu P, Wang Q, Xue J (2024). Association between personality type and patient-reported outcomes (PRO) in patients with atrial fibrillation. BMC cardiovascular disorders.

[33] Jia Z, Du X, Du J, Xia S, Guo L, Su X, Dong Z, Yuan Y, Zheng Y, Wu S, Guang X, Zhou X, Lin H, Cheng X, Dong J, Ma C (2021). Prevalence and factors associated with depressive and anxiety symptoms in a Chinese population with and without cardiovascular diseases. Journal of affective disorders.

[34] Teppo K, Jaakkola J, Biancari F, Halminen O, Putaala J, Mustonen P, Haukka J, Linna M, Kinnunen J, Tiili P, Kouki E, Penttilä T, Hartikainen J, Aro AL, Airaksinen KEJ, Lehto M (2022). Mental health conditions and risk of first-ever ischaemic stroke and death in patients with incident atrial fibrillation: a nationwide cohort study. European journal of clinical investigation.

[35] Hagengaard L, Polcwiartek C, Andersen MP, Sessa M, Krogager ML, Gislason G, Schou M, Torp-Pedersen C, Søgaard P, Kragholm KH (2021). Incident atrial fibrillation and risk of psychoactive drug redemptions and psychiatric hospital contacts: a Danish Nationwide Register-based Follow-up Study. European heart journal. Quality of care & clinical outcomes.

[36] Suša R, Ratinac M, Ćupurdija V, Novković L, Milojević-Ilić M, Petrović M, Igrutinović N, Vuleta M, Timotijević L, Kostić O, Čekerevac I (2023). Implementation of the baveno classification in obstructive sleep apnea and its correlation with symptoms of anxiety and depression. Medicina (Kaunas, Lithuania).

[37] Onishi N, Kyo S, Oi M, Jinnai T, Kuroda M, Shimizu Y, Imamura S, Harita T, Nishiuchi S, Hanazawa K, Tamura T, Izumi C, Nakagawa Y, Kaitani K (2021). Improvement in quality of life and cardiac function after catheter ablation for asymptomatic persistent atrial fibrillation. Journal of arrhythmia.

[38] Risom SS, Thygesen LC, Rasmussen TB, Borregaard B, Nørgaard MW, Mols R, Christensen AV, Thorup CB, Thrysoee L, Juel K, Ekholm O, Berg SK (2023). Association between risk factors and readmission for patients with atrial fibrillation treated with catheter ablation: results from the nationwide denheart study. The Journal of cardiovascular nursing.

[39] Zeng R, Pu X, Chen S, Chen C, Chen Y, Chen W, Fu H (2023). Oropharynx pain, discomfort, and economic impact of transesophageal echocardiography for planned radio-frequency catheter ablation in patients with atrial fibrillation: A cross-sectional survey study. International journal of cardiology. Heart & vasculature.

[40] Yu H, Zhao Y, Li Y (2022). Analysis of early warning diagnostic indexes and influencing factors of anxiety and depression in patients with arrhythmia. Evidence-based complementary and alternative medicine: eCAM.

[41] Ahn HJ, Lee SR, Choi EK, Bae NY, Ahn HJ, Kwon S, Lee SW, Han KD, Oh S, Lip GYH (2023). Increased risk of incident atrial fibrillation in young adults with mental disorders: a nationwide population-based study. Heart rhythm.

[42] Le Grande MR, Salvacion M, Shwaita L, Murphy BM, Jackson AC, Alvarenga ME (2024). Does coping style mediate the relationship between knowledge and psychosocial outcomes in women with atrial fibrillation?. Frontiers in psychiatry.

[43] Samuel M, Khairy P, Champagne J, Deyell MW, Macle L, Leong-Sit P, Novak P, Badra-Verdu M, Sapp J, Tardif JC, Andrade JG (2021). Association of atrial fibrillation burden with health-related quality of life after atrial fibrillation ablation: substudy of the cryoballoon vs contact-force atrial fibrillation ablation (CIRCA-DOSE) randomized clinical trial. JAMA cardiology.

[44] Zylla MM, Özdemir B, Hochadel M, Zeymer U, Akin I, Grau A, Schneider S, Alonso A, Waldecker B, Süselbeck T, Schwacke H, Haass M, Zahn R, Borggrefe M, Senges J, Frey N, Thomas D (2025). Community-based analysis of stroke prevention and effect of public interventions in atrial fibrillation: results from the ARENA project. Clinical research in cardiology: official journal of the German Cardiac Society.

[45] Wei W, Ma C, Tian Z, Bu M, Liu W, Song M (2024). Impact of early rehabilitation nursing on postoperative cardiac function and quality of life in patients with atrial fibrillation. Altern Ther Health Med.

[46] Dossett ML, Needles EW, Donahue Z, Gadenne G, Macklin EA, Ruskin JN, Denninger JW (2021). A SMART approach to reducing paroxysmal atrial fibrillation symptoms: Results from a pilot randomized controlled trial. Heart rhythm O2.

[47] Sedney C, Abu HO, Trymbulak K, Mehawej J, Wang Z, Waring ME, Saczynski J, McManus DD (2021). Sociodemographic, behavioral, and clinical factors associated with low atrial fibrillation knowledge among older adults with atrial fibrillation: The SAGE-AF study. Patient education and counseling.

[48] Sharma G, Mooventhan A, Naik G, Nivethitha L (2021). A review on role of yoga in the management of patients with cardiac arrhythmias. International journal of yoga.

[49] Minjie Z, Zhijuan X, Xinxin S, Shan Q (2024). Mediating effect of coping strategy and psychological status between illness perception and quality of life among patients with atrial fibrillation: a cross-sectional study. BMC cardiovascular disorders.

[50] Liu TH, Wu JY, Huang PY, Hsu WH, Chuang MH, Tsai YW, Hsieh KY, Lai CC (2025). Association between catheter ablation and psychiatric disorder risk in adults with atrial fibrillation: a multi-institutional retrospective cohort study. Frontiers in psychiatry.

[51] Zhang M, Chen J, Gao C, Gu S, Yang P, Xu Z (2022). Application value of whole-course health management for patients with nonvalvular atrial fibrillation with oral warfarin treatment. Am J Transl Res.

[52] Kamenskaya OV, Klinkova AS, Loginova IY, Porotnikova SS, Lomivorotov VN, Alsov SA, Sirota DA, Chernyavskiy AM (2024). Anxiety-depressive disorders in patients before and in the long terms after aortic replacement. Zhurnal nevrologii i psikhiatrii imeni S.S. Korsakova.

[53] Bae NY, Lee SR, Choi EK, Ahn HJ, Ahn HJ, Kwon S, Han KD, Lee KN, Oh S, Lip GYH (2022). Impact of mental disorders on the risk of atrial fibrillation in patients with diabetes mellitus: a nationwide population-based study. Cardiovascular diabetology.

[54] Silva LA, Silva KR, Saucedo SCM, Costa R (2024). Predictors of quality of life, anxiety and acceptance in patients with implantable cardioverter-defibrillator. Arquivos brasileiros de cardiologia.

[55] Cao Z, Hou Y, Yang H, Huang X, Wang X, Xu C (2023). Healthy sleep patterns and common mental disorders among individuals with cardiovascular disease: a prospective cohort study. Journal of affective disorders.

[56] Mehawej J, Tran KT, Filippaios A, Paul T, Abu HO, Ding E, Mishra A, Dai Q, Hariri E, Wilson SHoward, Asaker JC, Mathew J, Naeem S, Otabil EMensah, Soni A, McManus DD (2023). Self-reported efficacy in patient-physician interaction in relation to anxiety, patient activation, and health-related quality of life among stroke survivors. Annals of medicine.

[57] Mathew J, Mehawej J, Wang Z, Orwig T, Ding E, Filippaios A, Naeem S, Otabil EM, Hamel A, Noorishirazi K, Radu I, Saczynski J, McManus DD, Tran KV (2024). Health behavior outcomes in stroke survivors prescribed wearables for atrial fibrillation detection stratified by age. Journal of geriatric cardiology: JGC.

[58] Liu SY, Hsu YL, Tu YC, Lin CH, Wang SC, Lee YW, Shih YT, Chou MC, Lin CM (2022). Functional outcome prediction of ischemic stroke patients with atrial fibrillation accepting post-acute care training. Frontiers in neurology.

[59] Jaakkola J, Teppo K, Biancari F, Halminen O, Putaala J, Mustonen P, Haukka J, Linna M, Kinnunen J, Tiili P, Aro AL, Hartikainen J, Airaksinen KEJ, Lehto M (2022). The effect of mental health conditions on the use of oral anticoagulation therapy in patients with atrial fibrillation: the FinACAF study. European heart journal. Quality of care & clinical outcomes.

[60] Mensah Otabil E, Dai Q, Anzenberg P, Filippaios A, Ding E, Mehawej J, Mathew JE, Lessard D, Wang Z, Noorishirazi K, Hamel A, Paul T, Di Mezza D, Han D, Mohagheghian F, Soni A, Lin H, Barton B, Saczynski J, Chon KH, Tran KV, McManus DD (2023). Technology engagement is associated with higher perceived physical well-being in stroke patients prescribed smartwatches for atrial fibrillation detection. Frontiers in digital health.

[61] Gabilondo M, Loza J, Pereda A, Caballero O, Zamora N, Gorostiza A, Mar J (2021). Quality of life in patients with nonvalvular atrial fibrillation treated with oral anticoagulants. Hematology (Amsterdam, Netherlands).

[62] Mookerjee N, Schmalbach N, Antinori G, Thampi S, Windle-Puente D, Gilligan A, Huy H, Andrews M, Sun A, Gandhi R, Benedict W, Chang A, Sanders B, Nguyen J, Keesara MR, Aliev J, Patel A, Hughes I, Millstein I, Hunter K, Roy S (2023). Comorbidities and risk factors associated with insomnia in the elderly population. Journal of primary care & community health.

[63] Chu G, Calvert P, Sidhu B, Mavilakandy A, Kotb A, Tovmassian L, Kozhuharov N, Biermé C, Denham N, Pius C, O’Brien J, Ding WY, Luther V, Snowdon RL, Ng GA, Gupta D (2023). Patient experience of very high power short duration radiofrequency ablation for atrial fibrillation under mild conscious sedation. Journal of interventional cardiac electrophysiology: an international journal of arrhythmias and pacing.

[64] Rimmele DL, Schrage T, Lebherz L, Kriston L, Gerloff C, Härter M, Thomalla G Profiles of patients’ self-reported health after acute stroke. Neurological research and practice.

[65] Holmlund L, Hörnsten C, Hörnsten Å, Olsson K, Valham F, Hellström Ängerud K (2024). More positive patient-reported outcomes in patients newly diagnosed with atrial fibrillation: a comparative longitudinal study. European journal of cardiovascular nursing.

[66] Dokoupil J, Hrečko J, Čermáková E, Adamcová M, Pudil R (2022). Characteristics and outcomes of patients admitted for acute heart failure in a single-centre study. ESC heart failure.

[67] Naeem S, Jones T, Daniel J, Mehawej J, Filippaios A, Paul T, Wang Z, Howard-Wilson S, Lessard D, Ding E, Otabil EM, Noorishirazi K, Soni A, Saczynski J, Tran KV, McManus D (2024). Income in relation to psychosocial factors among stroke survivors using smartwatches for atrial fibrillation monitoring. Cardiology and cardiovascular medicine.

[68] Mihas P, Rosman L, Armbruster T, Walker J, Deyo Z, Gehi A (2024). Assessing a virtual education intervention for patients with atrial fibrillation: a qualitative study of patient perceptions. The Journal of cardiovascular nursing.

[69] Wu YW, Lin TH, Yang YP, Wu WT, Tu CM, Huang HK, Chu CY, Huang CC, Chien SC, Jhuo SJ, Chen CP (2025). Impact of shared decision-making in Taiwanese patients with atrial fibrillation eligible for novel oral anticoagulant therapy. Journal of the Formosan Medical Association Taiwan yi zhi.

[70] Hamilton E, Shone L, Reynolds C, Wu J, Nadarajah R, Gale C (2025). Perceptions of healthcare professionals on the use of a risk prediction model to inform atrial fibrillation screening: qualitative interview study in English primary care. BMJ open.

[71] de Hartog Keyzer JML, Pedersen SS, El Messaoudi S, Nijveldt R, Pop VJM (2022). Psychological distress is independently related to new coronary events at 8 years’ follow-up in elderly primary care patients with hypertension. Journal of psychosomatic research.

[72] Urbonas G, Raila G, Serapinas D, Valius L, Veličkiene D, Plisiene J, Vencevičiene L, Jurevičiene E, Liseckiene I (2023). Evaluation of satisfaction with healthcare services in multimorbid patients using PACIC+ questionnaire: a cross-sectional study. Medicina (Kaunas, Lithuania).

[73] Filippaios A, Tran KT, Mehawej J, Ding E, Paul T, Lessard D, Barton B, Lin H, Naeem S, Otabil EM, Noorishirazi K, Dai Q, Sadiq H, Chon KH, Soni A, Saczynski J, McManus DD (2022). Psychosocial measures in relation to smartwatch alerts for atrial fibrillation detection. Cardiovascular digital health journal.

[74] Waller A, Fakes K, Carey M, Dizon J, Parrey K, Coad M, Sanson-Fisher R (2023). Quality of life and mood disorders of mild to moderate stroke survivors in the early post-hospital discharge phase: a cross-sectional survey study. BMC psychology.

[75] Klein A, Cremer P, Kontzias A, Furqan M, Tubman R, Roy M, Lim-Watson MZ, Magestro M (2021). US database study of clinical burden and unmet need in recurrent pericarditis. Journal of the American Heart Association.

[76] Wawruch M, Murin J, Tesar T, Paduchova M, Petrova M, Celovska D, Havelkova B, Trnka M, Aarnio E (2021). Non-persistence with antiplatelet medications among older patients with peripheral arterial disease. Frontiers in pharmacology.

[77] Pemmasani SK, S RG, S V, Bhattacharyya R, Patel C, Gupta AK, Acharya A (2024). Genetic variants associated with longevity in long-living Indians. npj aging.

[78] Hsu PW, Shen YW, Syam S, Liang WM, Wu TN, Hsu JT, Fuh LJ (2022). Patients with periodontitis are at a higher risk of stroke: a Taiwanese cohort study. Journal of the Chinese Medical Association: JCMA.

[79] Kawamura C, Bhaskaran K, Konishi T, Sagara Y, Bando H, Shinozaki T, Nojiri S, Adomi M, Wong AYS, Tamiya N, Iwagami M (2025). Non-cancer risks among female breast cancer survivors: a matched cohort study in Japan. The Lancet regional health. Western Pacific. Western Pacific.

[80] Carnlöf C, Schenck-Gustafsson K, Jensen-Urstad M, Insulander P (2021). Instant electrocardiogram feedback with a new digital technique reduces symptoms caused by palpitations and increases health-related quality of life (the RedHeart study). European journal of cardiovascular nursing.

[81] Kovoor JG, McIntyre D, Chik WWB, Chow CK, Thiagalingam A (2021). Clinician-created educational video resources for shared decision-making in the outpatient management of chronic disease: development and evaluation Study. Journal of medical Internet research.

[82] Downer MB, Luengo-Fernandez R, Binney LE, Gutnikov S, Silver LE, McColl A, Rothwell PM (2024). Association of multimorbidity with mortality after stroke stratified by age, severity, etiology, and prior disability. International journal of stroke: official journal of the International Stroke Society.

[83] Harrison SL, Lane DA, Buckley BJR, Chatterjee K, Alobaida M, Shipley E, Lip GYH (2022). The liverpool heart and brain project (L-HARP): protocol for an observational cohort study of cardiovascular risk and outcomes following stroke. Vascular health and risk management.

[84] Kilkenny MF, Dalli LL, Sanders A, Olaiya MT, Kim J, Ung D, Andrew NE (2024). Comparison of comorbidities of stroke collected in administrative data, surveys, clinical trials and cohort studies. Health information management: journal of the Health Information Management Association of Australia.

